# Supplementary material for: Resolving the full spectrum of human genome variation using Linked-Reads
Source: Genome Res. 2019 Apr;29(4):635–45. doi: 10.1101/gr.234443.118 (PMC6442396; doi:10.1101/gr.234443.118)
Supplement: Supplemental Material [file supp_gr.234443.118_Supplemental_longranger-2.2.zip › longranger-2.2.tar.bz2!/lr2.2-public/lib/stan/cmdstan/stan/doc/api/html/classstan_1_1optimization_1_1_b_f_g_s_minimizer.html]

Stan: stan::optimization::BFGSMinimizer< FunctorType, QNUpdateType, Scalar, DimAtCompile > Class Template Reference


|  |  |
| --- | --- |
|  | Stan  2.5.0  probability, sampling & optimization |


- Main Page
- Related Pages
- Namespaces
- Classes
- Files

- Class List
- Class Index
- Class Hierarchy
- Class Members

All Classes Namespaces Files Functions Variables Typedefs Enumerations Enumerator Friends Macros Pages

- stan
- optimization
- BFGSMinimizer

Public Types |
Public Member Functions |
Public Attributes |
Protected Attributes |
List of all members

stan::optimization::BFGSMinimizer< FunctorType, QNUpdateType, Scalar, DimAtCompile > Class Template Reference

`#include <bfgs.hpp>`

|  |  |
| --- | --- |
| Public Types | |
| typedef Eigen::Matrix< Scalar,   DimAtCompile, 1 > | VectorT |
|  | |
| typedef Eigen::Matrix< Scalar,   DimAtCompile, DimAtCompile > | HessianT |
|  | |

|  |  |
| --- | --- |
| Public Member Functions | |
| QNUpdateType & | get\_qnupdate () |
|  | |
| const QNUpdateType & | get\_qnupdate () const |
|  | |
| const Scalar & | curr\_f () const |
|  | |
| const VectorT & | curr\_x () const |
|  | |
| const VectorT & | curr\_g () const |
|  | |
| const VectorT & | curr\_p () const |
|  | |
| const Scalar & | prev\_f () const |
|  | |
| const VectorT & | prev\_x () const |
|  | |
| const VectorT & | prev\_g () const |
|  | |
| const VectorT & | prev\_p () const |
|  | |
| Scalar | prev\_step\_size () const |
|  | |
| Scalar | rel\_grad\_norm () const |
|  | |
| Scalar | rel\_obj\_decrease () const |
|  | |
| const Scalar & | alpha0 () const |
|  | |
| const Scalar & | alpha () const |
|  | |
| const size\_t | iter\_num () const |
|  | |
| const std::string & | note () const |
|  | |
| std::string | get\_code\_string (int retCode) |
|  | |
|  | BFGSMinimizer (FunctorType &f) |
|  | |
| void | initialize (const VectorT &x0) |
|  | |
| int | step () |
|  | |
| int | minimize (VectorT &x0) |
|  | |

|  |  |
| --- | --- |
| Public Attributes | |
| LSOptions< Scalar > | \_ls\_opts |
|  | |
| ConvergenceOptions< Scalar > | \_conv\_opts |
|  | |

|  |  |
| --- | --- |
| Protected Attributes | |
| FunctorType & | \_func |
|  | |
| VectorT | \_gk |
|  | |
| VectorT | \_gk\_1 |
|  | |
| VectorT | \_xk\_1 |
|  | |
| VectorT | \_xk |
|  | |
| VectorT | \_pk |
|  | |
| VectorT | \_pk\_1 |
|  | |
| Scalar | \_fk |
|  | |
| Scalar | \_fk\_1 |
|  | |
| Scalar | \_alphak\_1 |
|  | |
| Scalar | \_alpha |
|  | |
| Scalar | \_alpha0 |
|  | |
| size\_t | \_itNum |
|  | |
| std::string | \_note |
|  | |
| QNUpdateType | \_qn |
|  | |

## Detailed Description

### template<typename FunctorType, typename QNUpdateType, typename Scalar = double, int DimAtCompile = Eigen::Dynamic> class stan::optimization::BFGSMinimizer< FunctorType, QNUpdateType, Scalar, DimAtCompile >

Definition at line 73 of file bfgs.hpp.

## Member Typedef Documentation

template<typename FunctorType, typename QNUpdateType, typename Scalar = double, int DimAtCompile = Eigen::Dynamic>

typedef Eigen::Matrix<Scalar,DimAtCompile,DimAtCompile> stan::optimization::BFGSMinimizer< FunctorType, QNUpdateType, Scalar, DimAtCompile >::HessianT

Definition at line 76 of file bfgs.hpp.

template<typename FunctorType, typename QNUpdateType, typename Scalar = double, int DimAtCompile = Eigen::Dynamic>

typedef Eigen::Matrix<Scalar,DimAtCompile,1> stan::optimization::BFGSMinimizer< FunctorType, QNUpdateType, Scalar, DimAtCompile >::VectorT

Definition at line 75 of file bfgs.hpp.

## Constructor & Destructor Documentation

template<typename FunctorType, typename QNUpdateType, typename Scalar = double, int DimAtCompile = Eigen::Dynamic>

|  |  |  |  |  |  |
| --- | --- | --- | --- | --- | --- |
| stan::optimization::BFGSMinimizer< FunctorType, QNUpdateType, Scalar, DimAtCompile >::BFGSMinimizer | ( | FunctorType & | *f* | ) |  |

inline

Definition at line 143 of file bfgs.hpp.

## Member Function Documentation

template<typename FunctorType, typename QNUpdateType, typename Scalar = double, int DimAtCompile = Eigen::Dynamic>

|  |  |  |  |  |
| --- | --- | --- | --- | --- |
| const Scalar& stan::optimization::BFGSMinimizer< FunctorType, QNUpdateType, Scalar, DimAtCompile >::alpha | ( |  | ) | const |

inline

Definition at line 115 of file bfgs.hpp.

template<typename FunctorType, typename QNUpdateType, typename Scalar = double, int DimAtCompile = Eigen::Dynamic>

|  |  |  |  |  |
| --- | --- | --- | --- | --- |
| const Scalar& stan::optimization::BFGSMinimizer< FunctorType, QNUpdateType, Scalar, DimAtCompile >::alpha0 | ( |  | ) | const |

inline

Definition at line 114 of file bfgs.hpp.

template<typename FunctorType, typename QNUpdateType, typename Scalar = double, int DimAtCompile = Eigen::Dynamic>

|  |  |  |  |  |
| --- | --- | --- | --- | --- |
| const Scalar& stan::optimization::BFGSMinimizer< FunctorType, QNUpdateType, Scalar, DimAtCompile >::curr\_f | ( |  | ) | const |

inline

Definition at line 94 of file bfgs.hpp.

template<typename FunctorType, typename QNUpdateType, typename Scalar = double, int DimAtCompile = Eigen::Dynamic>

|  |  |  |  |  |
| --- | --- | --- | --- | --- |
| const VectorT& stan::optimization::BFGSMinimizer< FunctorType, QNUpdateType, Scalar, DimAtCompile >::curr\_g | ( |  | ) | const |

inline

Definition at line 96 of file bfgs.hpp.

template<typename FunctorType, typename QNUpdateType, typename Scalar = double, int DimAtCompile = Eigen::Dynamic>

|  |  |  |  |  |
| --- | --- | --- | --- | --- |
| const VectorT& stan::optimization::BFGSMinimizer< FunctorType, QNUpdateType, Scalar, DimAtCompile >::curr\_p | ( |  | ) | const |

inline

Definition at line 97 of file bfgs.hpp.

template<typename FunctorType, typename QNUpdateType, typename Scalar = double, int DimAtCompile = Eigen::Dynamic>

|  |  |  |  |  |
| --- | --- | --- | --- | --- |
| const VectorT& stan::optimization::BFGSMinimizer< FunctorType, QNUpdateType, Scalar, DimAtCompile >::curr\_x | ( |  | ) | const |

inline

Definition at line 95 of file bfgs.hpp.

template<typename FunctorType, typename QNUpdateType, typename Scalar = double, int DimAtCompile = Eigen::Dynamic>

|  |  |  |  |  |  |
| --- | --- | --- | --- | --- | --- |
| std::string stan::optimization::BFGSMinimizer< FunctorType, QNUpdateType, Scalar, DimAtCompile >::get\_code\_string | ( | int | *retCode* | ) |  |

inline

Definition at line 120 of file bfgs.hpp.

template<typename FunctorType, typename QNUpdateType, typename Scalar = double, int DimAtCompile = Eigen::Dynamic>

|  |  |  |  |  |
| --- | --- | --- | --- | --- |
| QNUpdateType& stan::optimization::BFGSMinimizer< FunctorType, QNUpdateType, Scalar, DimAtCompile >::get\_qnupdate | ( |  | ) |  |

inline

Definition at line 91 of file bfgs.hpp.

template<typename FunctorType, typename QNUpdateType, typename Scalar = double, int DimAtCompile = Eigen::Dynamic>

|  |  |  |  |  |
| --- | --- | --- | --- | --- |
| const QNUpdateType& stan::optimization::BFGSMinimizer< FunctorType, QNUpdateType, Scalar, DimAtCompile >::get\_qnupdate | ( |  | ) | const |

inline

Definition at line 92 of file bfgs.hpp.

template<typename FunctorType, typename QNUpdateType, typename Scalar = double, int DimAtCompile = Eigen::Dynamic>

|  |  |  |  |  |  |
| --- | --- | --- | --- | --- | --- |
| void stan::optimization::BFGSMinimizer< FunctorType, QNUpdateType, Scalar, DimAtCompile >::initialize | ( | const VectorT & | *x0* | ) |  |

inline

Definition at line 145 of file bfgs.hpp.

template<typename FunctorType, typename QNUpdateType, typename Scalar = double, int DimAtCompile = Eigen::Dynamic>

|  |  |  |  |  |
| --- | --- | --- | --- | --- |
| const size\_t stan::optimization::BFGSMinimizer< FunctorType, QNUpdateType, Scalar, DimAtCompile >::iter\_num | ( |  | ) | const |

inline

Definition at line 116 of file bfgs.hpp.

template<typename FunctorType, typename QNUpdateType, typename Scalar = double, int DimAtCompile = Eigen::Dynamic>

|  |  |  |  |  |  |
| --- | --- | --- | --- | --- | --- |
| int stan::optimization::BFGSMinimizer< FunctorType, QNUpdateType, Scalar, DimAtCompile >::minimize | ( | VectorT & | *x0* | ) |  |

inline

Definition at line 276 of file bfgs.hpp.

template<typename FunctorType, typename QNUpdateType, typename Scalar = double, int DimAtCompile = Eigen::Dynamic>

|  |  |  |  |  |
| --- | --- | --- | --- | --- |
| const std::string& stan::optimization::BFGSMinimizer< FunctorType, QNUpdateType, Scalar, DimAtCompile >::note | ( |  | ) | const |

inline

Definition at line 118 of file bfgs.hpp.

template<typename FunctorType, typename QNUpdateType, typename Scalar = double, int DimAtCompile = Eigen::Dynamic>

|  |  |  |  |  |
| --- | --- | --- | --- | --- |
| const Scalar& stan::optimization::BFGSMinimizer< FunctorType, QNUpdateType, Scalar, DimAtCompile >::prev\_f | ( |  | ) | const |

inline

Definition at line 99 of file bfgs.hpp.

template<typename FunctorType, typename QNUpdateType, typename Scalar = double, int DimAtCompile = Eigen::Dynamic>

|  |  |  |  |  |
| --- | --- | --- | --- | --- |
| const VectorT& stan::optimization::BFGSMinimizer< FunctorType, QNUpdateType, Scalar, DimAtCompile >::prev\_g | ( |  | ) | const |

inline

Definition at line 101 of file bfgs.hpp.

template<typename FunctorType, typename QNUpdateType, typename Scalar = double, int DimAtCompile = Eigen::Dynamic>

|  |  |  |  |  |
| --- | --- | --- | --- | --- |
| const VectorT& stan::optimization::BFGSMinimizer< FunctorType, QNUpdateType, Scalar, DimAtCompile >::prev\_p | ( |  | ) | const |

inline

Definition at line 102 of file bfgs.hpp.

template<typename FunctorType, typename QNUpdateType, typename Scalar = double, int DimAtCompile = Eigen::Dynamic>

|  |  |  |  |  |
| --- | --- | --- | --- | --- |
| Scalar stan::optimization::BFGSMinimizer< FunctorType, QNUpdateType, Scalar, DimAtCompile >::prev\_step\_size | ( |  | ) | const |

inline

Definition at line 103 of file bfgs.hpp.

template<typename FunctorType, typename QNUpdateType, typename Scalar = double, int DimAtCompile = Eigen::Dynamic>

|  |  |  |  |  |
| --- | --- | --- | --- | --- |
| const VectorT& stan::optimization::BFGSMinimizer< FunctorType, QNUpdateType, Scalar, DimAtCompile >::prev\_x | ( |  | ) | const |

inline

Definition at line 100 of file bfgs.hpp.

template<typename FunctorType, typename QNUpdateType, typename Scalar = double, int DimAtCompile = Eigen::Dynamic>

|  |  |  |  |  |
| --- | --- | --- | --- | --- |
| Scalar stan::optimization::BFGSMinimizer< FunctorType, QNUpdateType, Scalar, DimAtCompile >::rel\_grad\_norm | ( |  | ) | const |

inline

Definition at line 105 of file bfgs.hpp.

template<typename FunctorType, typename QNUpdateType, typename Scalar = double, int DimAtCompile = Eigen::Dynamic>

|  |  |  |  |  |
| --- | --- | --- | --- | --- |
| Scalar stan::optimization::BFGSMinimizer< FunctorType, QNUpdateType, Scalar, DimAtCompile >::rel\_obj\_decrease | ( |  | ) | const |

inline

Definition at line 108 of file bfgs.hpp.

template<typename FunctorType, typename QNUpdateType, typename Scalar = double, int DimAtCompile = Eigen::Dynamic>

|  |  |  |  |  |
| --- | --- | --- | --- | --- |
| int stan::optimization::BFGSMinimizer< FunctorType, QNUpdateType, Scalar, DimAtCompile >::step | ( |  | ) |  |

inline

Definition at line 158 of file bfgs.hpp.

## Member Data Documentation

template<typename FunctorType, typename QNUpdateType, typename Scalar = double, int DimAtCompile = Eigen::Dynamic>

Scalar stan::optimization::BFGSMinimizer< FunctorType, QNUpdateType, Scalar, DimAtCompile >::\_alpha

protected

Definition at line 82 of file bfgs.hpp.

template<typename FunctorType, typename QNUpdateType, typename Scalar = double, int DimAtCompile = Eigen::Dynamic>

Scalar stan::optimization::BFGSMinimizer< FunctorType, QNUpdateType, Scalar, DimAtCompile >::\_alpha0

protected

Definition at line 82 of file bfgs.hpp.

template<typename FunctorType, typename QNUpdateType, typename Scalar = double, int DimAtCompile = Eigen::Dynamic>

Scalar stan::optimization::BFGSMinimizer< FunctorType, QNUpdateType, Scalar, DimAtCompile >::\_alphak\_1

protected

Definition at line 81 of file bfgs.hpp.

template<typename FunctorType, typename QNUpdateType, typename Scalar = double, int DimAtCompile = Eigen::Dynamic>

ConvergenceOptions<Scalar> stan::optimization::BFGSMinimizer< FunctorType, QNUpdateType, Scalar, DimAtCompile >::\_conv\_opts

Definition at line 89 of file bfgs.hpp.

template<typename FunctorType, typename QNUpdateType, typename Scalar = double, int DimAtCompile = Eigen::Dynamic>

Scalar stan::optimization::BFGSMinimizer< FunctorType, QNUpdateType, Scalar, DimAtCompile >::\_fk

protected

Definition at line 81 of file bfgs.hpp.

template<typename FunctorType, typename QNUpdateType, typename Scalar = double, int DimAtCompile = Eigen::Dynamic>

Scalar stan::optimization::BFGSMinimizer< FunctorType, QNUpdateType, Scalar, DimAtCompile >::\_fk\_1

protected

Definition at line 81 of file bfgs.hpp.

template<typename FunctorType, typename QNUpdateType, typename Scalar = double, int DimAtCompile = Eigen::Dynamic>

FunctorType& stan::optimization::BFGSMinimizer< FunctorType, QNUpdateType, Scalar, DimAtCompile >::\_func

protected

Definition at line 79 of file bfgs.hpp.

template<typename FunctorType, typename QNUpdateType, typename Scalar = double, int DimAtCompile = Eigen::Dynamic>

VectorT stan::optimization::BFGSMinimizer< FunctorType, QNUpdateType, Scalar, DimAtCompile >::\_gk

protected

Definition at line 80 of file bfgs.hpp.

template<typename FunctorType, typename QNUpdateType, typename Scalar = double, int DimAtCompile = Eigen::Dynamic>

VectorT stan::optimization::BFGSMinimizer< FunctorType, QNUpdateType, Scalar, DimAtCompile >::\_gk\_1

protected

Definition at line 80 of file bfgs.hpp.

template<typename FunctorType, typename QNUpdateType, typename Scalar = double, int DimAtCompile = Eigen::Dynamic>

size\_t stan::optimization::BFGSMinimizer< FunctorType, QNUpdateType, Scalar, DimAtCompile >::\_itNum

protected

Definition at line 83 of file bfgs.hpp.

template<typename FunctorType, typename QNUpdateType, typename Scalar = double, int DimAtCompile = Eigen::Dynamic>

LSOptions<Scalar> stan::optimization::BFGSMinimizer< FunctorType, QNUpdateType, Scalar, DimAtCompile >::\_ls\_opts

Definition at line 88 of file bfgs.hpp.

template<typename FunctorType, typename QNUpdateType, typename Scalar = double, int DimAtCompile = Eigen::Dynamic>

std::string stan::optimization::BFGSMinimizer< FunctorType, QNUpdateType, Scalar, DimAtCompile >::\_note

protected

Definition at line 84 of file bfgs.hpp.

template<typename FunctorType, typename QNUpdateType, typename Scalar = double, int DimAtCompile = Eigen::Dynamic>

VectorT stan::optimization::BFGSMinimizer< FunctorType, QNUpdateType, Scalar, DimAtCompile >::\_pk

protected

Definition at line 80 of file bfgs.hpp.

template<typename FunctorType, typename QNUpdateType, typename Scalar = double, int DimAtCompile = Eigen::Dynamic>

VectorT stan::optimization::BFGSMinimizer< FunctorType, QNUpdateType, Scalar, DimAtCompile >::\_pk\_1

protected

Definition at line 80 of file bfgs.hpp.

template<typename FunctorType, typename QNUpdateType, typename Scalar = double, int DimAtCompile = Eigen::Dynamic>

QNUpdateType stan::optimization::BFGSMinimizer< FunctorType, QNUpdateType, Scalar, DimAtCompile >::\_qn

protected

Definition at line 85 of file bfgs.hpp.

template<typename FunctorType, typename QNUpdateType, typename Scalar = double, int DimAtCompile = Eigen::Dynamic>

VectorT stan::optimization::BFGSMinimizer< FunctorType, QNUpdateType, Scalar, DimAtCompile >::\_xk

protected

Definition at line 80 of file bfgs.hpp.

template<typename FunctorType, typename QNUpdateType, typename Scalar = double, int DimAtCompile = Eigen::Dynamic>

VectorT stan::optimization::BFGSMinimizer< FunctorType, QNUpdateType, Scalar, DimAtCompile >::\_xk\_1

protected

Definition at line 80 of file bfgs.hpp.

---

The documentation for this class was generated from the following file:

- src/stan/optimization/bfgs.hpp


---

[ Stan Home Page ]

*© 2011–2014,
Stan Development Team.*
